# Trolox, r-irisin and resveratrol cocktail to counteract osteoblast metabolism alterations in osteoarthritis and osteoporosis

**DOI:** 10.1007/s00774-025-01642-7

**Published:** 2025-09-16

**Authors:** Roberto Bonanni, Angela Falvino, Amarildo Smakaj, Lucia Tranquillo, Anna Maria Rinaldi, Giovanna D’Arcangelo, Pierangelo Cifelli, Virginia Tancredi, Ida Cariati, Umberto Tarantino

**Affiliations:** 1 Department of Biotechnological and Applied Clinical Sciences, University of L’Aquila, L’Aquila, Italy; 2 Department of Biomedicine and Prevention, “Tor Vergata” University of Rome, Rome, Italy; 3 Department of Orthopaedics and Traumatology, “Policlinico Tor Vergata” Foundation, Rome, Italy; 4 Department of Systems Medicine, “Tor Vergata” University of Rome, Rome, Italy; 5 Centre of Space Bio-Medicine, “Tor Vergata” University of Rome, Rome, Italy; 6 Catholic University “Our Lady of Good Counsel”, Tirana, Albania

**Keywords:** Osteoporosis, Osteoarthritis, Bone metabolism, Musculoskeletal aging, Physiology

## Abstract

**Introduction:**

Osteoarthritis and osteoporosis are age-related musculoskeletal disorders characterized by increased oxidative stress and cellular senescence, which contribute to altered metabolism and disease progression. Although research in this field is constantly evolving, the discovery of new molecular targets and drug combinations to counteract musculoskeletal disorders remains a goal of great interest. This study aimed to evaluate the efficacy of a cocktail of trolox, recombinant irisin (r-irisin) and resveratrol in modulation of osteoblastic metabolism by investigating the expression of NADPH oxidase 4 (NOX4), sirtuin 1 (SIRT1) and pentraxin 3 (PTX3).

**Materials and methods:**

20 male patients undergoing hip arthroplasty were enrolled, including ten patients with coxarthrosis and ten patients with osteoporosis. Femoral head biopsies were taken from each patient to isolate primary osteoblast cultures, which were treated with the cocktail for 6 days.

**Results:**

The cocktail of trolox, r-irisin and resveratrol increased cell viability, and reduced ROS and senescence β-galactosidase activity (SA-β-Gal) levels. In addition, western blotting analysis showed reduced expression of NOX4 and increased expression of SIRT1 and PTX3 in both experimental groups, although with more pronounced effects in osteoarthritic patients, highlighting lower treatment efficacy in the presence of osteoporosis.

**Conclusions:**

The improvement in cell viability and reduction in oxidative stress and cellular senescence observed through treatment-induced modulation of the NOX4–SIRT1 axis and PTX3 suggests a protective role for these biomarkers in bone metabolism. These findings could offer new perspectives in counteracting the effects of aging on the skeletal system by improving bone health and mitigating metabolic alterations.

**Supplementary Information:**

The online version contains supplementary material available at 10.1007/s00774-025-01642-7.

## Introduction

Aging is associated with numerous biological changes, including genomic instability, cellular senescence, and increased inflammation, affecting almost all organs and systems, promoting the development of chronic and/or degenerative diseases [1, 2]. In the musculoskeletal system, these changes can compromise the structure and metabolism of bone tissue, contributing to the onset of disorders, such as osteoarthritis and osteoporosis. These conditions increase the risk of functional limitations and sedentary lifestyle, leading to increased disability and mortality [3]. Although the pathological mechanisms underlying these diseases have been partly elucidated, factors such as oxidative stress and inflammation play a crucial role in their progression [4]. Particularly, impairment of the microarchitecture of subchondral bone is a common feature of both osteoporosis and the early stages of osteoarthritis, suggesting the possibility of developing integrated therapeutic strategies to counteract these age-related musculoskeletal disorders [5].

Several evidences have suggested that irisin, a hormone released by skeletal muscle in response to exercise, may be a promising strategy for the prevention of osteoarthritis and osteoporosis, due to its ability to stimulate bone formation and inhibit tissue resorption [6, 7]. In this context, Ostojic et al. reported that irisin can inhibit the nuclear factor kappa-light-chain enhancer of activated B cells (NF-κB) pathway in osteoarthritis, modulating the inflammatory response and reducing cartilage and bone tissue erosion through inhibition of osteoclastic differentiation [8]. Noteworthy, a potential therapeutic role of irisin has also been suggested in osteoporosis, as demonstrated by its ability to promote osteoblast differentiation, stimulate bone formation through regulation of the Wnt/β-catenin pathway, and promote mineralization through the expression of pentraxin 3 (PTX3), a novel biomarker of bone mineralization [9–11]. In this regard, human osteoblasts are known to express PTX3, the levels of which significantly affect bone deposition, with a reduction observed in disorders characterized by excessive bone resorption [12, 13].

Important benefits have also been observed following treatment of osteoblasts with trolox, a potent antioxidant analog of vitamin E [14]. Specifically, Morabito and colleagues demonstrated its efficacy in counteracting cell death in osteoblasts exposed to simulated microgravity, suggesting its potential use in the treatment of bone conditions characterized by no loading [15]. In addition, Lee et al. reported that trolox significantly inhibits osteoclast formation through inhibition of receptor activator of NF-κB ligand (RANKL)-mediated signaling, highlighting the ability of this antioxidant to counteract bone resorption [16].

Notably, the development of antioxidant-based strategies is a key element in therapies aimed at countering cellular aging [17, 18]. Indeed, reactive oxygen species (ROS) produced by NADPH oxidase 4 (NOX4) appear to play a crucial role in osteoblast metabolism. However, although NOX4 has been proposed as a promising therapeutic target for osteoarthritis and osteoporosis, its involvement in their pathogenesis needs further investigation [19–21].

A major role in bone homeostasis has also been attributed to sirtuin 1 (SIRT1), an NAD^+^-dependent deacetylase that is critical in the regulation of cellular metabolism and the balance between bone formation and resorption [22]. SIRT1 is known to enhance the viability of mesenchymal stem cells and osteoblasts, counteracting their senescence and promoting osteogenesis [23]. Not surprisingly, down-regulation of SIRT1 has been associated with decreased bone mineral density (BMD) and increased fragility, suggesting both its potential role as a biomarker of bone metabolism and a promising therapeutic target to counteract diseases characterized by excessive bone resorption [24].

Importantly, resveratrol, a polyphenol found in red grapes and berries, has been proposed to modulate SIRT1 activity by promoting its activation [25]. Specifically, resveratrol treatment dose-dependently increases SIRT1 expression in osteoblasts and significantly improves BMD in animal models of osteoporosis [26]. In agreement, Wong and colleagues conducted a randomized controlled trial on the effects of resveratrol supplementation on the bone health of postmenopausal women, observing improved BMD in the lumbar spine and femoral neck [27].

Although the effects of trolox, r-irisin and resveratrol on osteoblasts have been documented individually, there is no evidence regarding the consequences of combined treatment on these cells. A cocktail of these factors could, through simultaneous activation of different signaling pathways, enhance the mineralization capacity of osteoblasts by promoting PTX3 expression, reduce NOX4-mediated ROS production, and improve cellular metabolism through up-regulation of SIRT1. Therefore, this study aimed to evaluate the effects of a combined treatment with trolox, r-irisin and resveratrol on the metabolism of primary osteoblasts isolated from patients with osteoarthritis or osteoporosis, analyzing the cellular response in each pathological group. Particular attention was paid to the modulation of NOX4, SIRT1 and PTX3, to explore the regulatory mechanisms involved in bone physiology in two distinct clinical contexts characterized by alterations in bone metabolism.

## Materials and methods

### Participants

A total of 20 male patients admitted to the Department of Orthopedics and Traumatology at the “Policlinico Tor Vergata” Foundation were enrolled in this study and divided into two experimental groups: ten patients undergoing hip arthroplasty for osteoarthritis (OA) and ten patients undergoing hip arthroplasty for fragility fracture (OP).

Exclusion criteria included subjects with endocrine disorders of mineral and bone metabolism, chronic viral infections, myopathies or other neuromuscular diseases, diabetes, neoplasms, chronic corticosteroid administration for autoimmune diseases (more than 1 month), alcohol abuse, or previous orthopedic surgical implants.

### Clinical evaluation

Classification into OA and OP patients was conducted based on dual-energy X-ray absorptiometry (DXA), *T*-score and radiographic evaluation. Specifically, a Lunar DXA device (GE Healthcare, Madison, WI, USA) was used to measure BMD by DXA in each patient. In accordance with the manufacturer’s instructions, BMD was measured in grams per square centimeter with a coefficient of variation of 0.7% by scans of the lumbar spine (L1–L4) and femur (neck and total). Measurements were taken on the non-dominant side for OA patients, supine on an examination table with the limbs slightly abducted, while BMD was measured on the non-injured limb for OP patients. DXA assessment was performed 1 day before surgery for OA patients and 1 month after surgery for OP patients, expressing all results as *T*-scores.

In addition, radiographs of the hip were taken to investigate hip osteoarthritis. The evaluation was conducted by two orthopedists independently and at different times, using the Kellgren and Lawrence radiographic atlas (K–L) according to which all patients with a K–L grade ≥ 2 were considered osteoarthritic [28].

### Specimen collection

Biopsies of the femoral head from each patient were taken during hip arthroplasty surgery and used for subsequent qualitative and quantitative analyses. Each experimental procedure was performed according to the World Medical Association’s Code of Ethics (Declaration of Helsinki) and was conducted with the approval of the Lazio Area 2 Territorial Ethics Committee (CET) (approval reference number #25/23). Written informed consent was obtained from each patient before the surgical procedure.

### Isolation and culture of primary human osteoblastic cells

Primary osteoblast cultures were set up using trabecular bone fragments taken during hip arthroplasty surgery from each patient. As previously mentioned [11], the fragments were first washed in phosphate-buffered saline (PBS) and then incubated at 37 °C with 1 mg/mL porcine pancreatic trypsin ≥ 60 U/mg (SERVA Electrophoresis GmbH Heidelberg, DE) diluted in PBS. Then, the bone fragments were subjected to repeated digestions with 2.5 mg/mL collagenase NB 4G Proved grade ≥ 0.18 U/mg (SERVA Electrophoresis GmbH, Heidelberg, DE) diluted in PBS with calcium and magnesium. At the end of digestion, the supernatant was collected and centrifuged at 340 RCF for 10 min. Cells were seeded in a 24-well plate at a density of 2 × 10^4^ cells/well and maintained in DMEM-F12 (Biowest SAS, Nuaillé, France) supplemented with 10% fetal bovine serum (FBS) (Biowest SAS, Nuaillé, France), 100 units/mL penicillin and 100 μg/mL streptomycin (Sigma-Aldrich, St. Louis, MO, USA) and 2 mmol/L stable glutamine (Biowest SAS, Nuaillé, France) in a 37 °C, 5% CO_2_ incubator until confluence was reached. The culture medium was changed every 2–3 days.

### Primary cultures of human osteoblasts conditioned with trolox, r-irisin and resveratrol cocktail

Primary osteoblast cultures were treated with a cocktail of trolox, r-irisin and resveratrol to investigate their efficacy in preventing and/or counteracting cellular aging. Specifically, cells were seeded in a 24-well plate at a density of 2 × 10^4^ cells/well and were incubated with 1 × 10^–4^ M trolox (S-238815, Sigma Aldrich, St. Louis, MO, USA), 10 ng/mL r-irisin (AG-20B-0153, AdipoGen® Life Sciences, Liestal, Switzerland) and 2.5 × 10^–5^ M resveratrol (554,325, Sigma Aldrich, St. Louis, MO, USA) for 6 days. Subsequently, treated cell samples were subjected to the same experimental procedures as untreated cells. Importantly, the individual effects of each substance were evaluated in the preliminary stages of our research by treating primary osteoblast cultures with 1 × 10^–4^ M trolox, 10 ng/mL r-irisin, or 2.5 × 10^–5^ M resveratrol for 6 days and are shown in Figure S1.

### Immunocytochemistry

An immunocytochemistry analysis was performed investigating both alkaline phosphatase (ALP) expression to characterize primary osteoblast cultures and PTX3 expression to study the mineralization process. After fixation in 4% paraformaldehyde for 15 min, the cell samples were pre-treated with EDTA citrate (pH 7.8) for 30 min at 95 °C and then incubated for 1 h with rabbit polyclonal anti-ALP antibody (ab224335, AbCam, Cambridge, United Kingdom) or rat monoclonal anti-PTX3 antibody (clone MNB1, AbCam, Cambridge, United Kingdom). Washings were performed with PBS/Tween20 (pH 7.6) (UCS Diagnostic, Rome, Italy). The immunocytochemical reaction was detected using the horseradish peroxidase (HRP)-3,3′-diaminobenzidine (DAB) detection kit (UCS Diagnostic, Rome, Italy). Specifically, 50 μL of DAB/450 μL of substrate were incubated for 3 min. The immunostaining background was evaluated with negative controls for each reaction by incubating the sections with secondary antibodies (HRP) alone or with the detection system (DAB) alone (Figure S2).

PTX3 immunopositive cells were detected with NIS-Elements software (5.30.01; Laboratory Imaging, Prague, Czech Republic) and expressed as a percentage of the total analyzed for PTX3. For each condition, the experiment was conducted in triplicate (*n* = 12 from *N* = 4 experiments).

### Cell viability assessment

CellTiter 96 AQueous One (Promega, Madison, WI, USA), a colorimetric method incorporates a tetrazolium compound (3-(4,5-dimethylthiazol-2-yl)-5-(3-carboxymethoxyphenyl)-2-(4-sulfophenyl)-2H-tetrazolium-MTS) and an electron-coupling reagent (phenazinamethosulfate—PMS), was used to identify viable cells. As described above [29], 20 µL of MTS/PMS solution was added to 100 µL of Hank’s balanced salt solution (HBSS) in each well and incubated for at least 2 h at 37 °C. The final concentrations of MTS and PMS were 333 μg/mL and 25 μM, respectively. The conversion of MTS to soluble formazan in the culture medium generates a dye whose absorbance at 490 nm was measured directly in the 96-well assay plates with a microplate reader (Spark Multimode Microplate Reader-Tecan, Austria).

The absorbance represents a measure of cell viability and was used to investigate the possible toxicity point of the administered substances. For each condition, the experiment was conducted in quintuplicate (*n* = 25 from *N* = 5 experiments).

### Measurement of intracellular ROS level

Intracellular ROS levels were measured using the fluorescent probe 2’,7’-dichlorodihydrofluorescein di-acetate (H_2_DCFDA) (D399, Invitrogen™, ThermoFisher Scientific, USA). As described previously [30], cell samples were washed with PBS and incubated with 10 μM of H_2_DCFDA for 40 min at 37 °C in the dark. A microplate reader (Spark Multimode Microplate Reader-Tecan, Austria) was used to measure the average fluorescence intensity. For each condition, the experiment was conducted in quintuplicate (*n* = 25 from *N* = 5 experiments).

### Senescence β-galactosidase activity (SA-β-gal) assay

SA-β-gal quantification in primary osteoblast cultures was performed using the specific SA-β-gal assay kit (23,833, Cell Signaling Technology, Inc., Danvers, MA, United States), according to the experimental procedure [31]. Protein extraction was conducted using 1X senescent cell lysis buffer enriched with 1.0 mM phenylmethanesulfonylfluoride (PMSF) and a cocktail of protease and phosphatase inhibitors. After removal of the culture medium, cells were washed with 1X PBS, lysed with 100 μL of cold 1X lysis buffer, incubated on ice for 5 min and harvested by scraping. The lysate was homogenized and centrifuged at 20,817 RCF for 5 min at 4 °C.

In parallel, 2X assay buffer was prepared and combined with 50 μL of cell lysate in a 96-well plate, incubated at 37 °C, in the dark, for 1–3 h. Fluorescence intensity was measured at 360 nm excitation and 465 nm emission using a microplate reader (Spark Multimode Microplate Reader—Tecan, Austria). For each condition, the experiment was conducted in triplicate (*n* = 15 from *N* = 5 experiments).

### Western blotting analysis

The expressions of NOX4, SIRT1, and PTX3 in primary cultures of osteoblasts derived from OA and OP patients were measured by western blotting analysis. Briefly, proteins samples extracted using RIPA buffer were separated by 8–16% precast SDS–PAGE (Bio-Rad, Hercules, CA, United States) under reduced conditions. Protein concentration was determined using the Pierce BCA Protein Assay Kit (Thermo Scientific, Vilnius, Lithuania). Equal amounts of protein (20 μg) were resolved on 8–16% precast SDS–PAGE and transferred to PVDF membrane. Membranes were incubated with rabbit polyclonal anti-NOX4 antibody (BS6796, Bioworld Technology, Inc., United States), mouse monoclonal anti-SIRT1 antibody (ab110304, AbCam, Cambridge, United Kingdom), or rat monoclonal anti-PTX3 (clone MNB1, AbCam) and successively with anti-rabbit IgG coupled to HRP, anti-mouse IgG coupled to HRP, or anti-rat IgG coupled to HRP, respectively. Moreover, normalization was performed by incubating the same membranes with mouse monoclonal anti-GAPDH (ab8245, AbCam, Cambridge, United Kingdom). Immunoreactive electrophoretic bands were detected by enhanced chemiluminescence (ECL Advance, Amersham; GE Healthcare Life Sciences, Little Chalfont, Buckinghamshire, United Kingdom) using a VersaDoc 5000 Imager (Bio-Rad).

The expression levels of NOX4, SIRT1, and PTX3 under the different experimental conditions were quantified by calculating the densitometric values of the relevant bands and normalizing the results against those of GAPDH, expressing them as mean ± standard error. The original western blotting images are shown in Figure S3.

### Immunofluorescence

An immunofluorescence analysis was conducted to explore the potential co-expression of NOX4 and SIRT1 in primary cultures of osteoblasts derived from OA and OP patients. In detail, after fixation in 4% paraformaldehyde dissolved in 0.9% saline solution for 30 min, cell cultures were pre-treated with EDTA citrate, pH 7.8 for 20 min at 95 °C, and incubated for 1 h with rabbit polyclonal anti-NOX4 antibody (NB110-58849, Novus Biologicals, Littleton, CO, United States), or mouse monoclonal anti-SIRT1 antibody (ab110304, AbCam, Cambridge, United Kingdom). Reaction was revealed using secondary antibodies (A-11008, A-11004, Alexa Fluor® 488, Thermo Fisher Scientific, Waltham, MA USA). Washing was performed with PBS/Tween20 pH 7.6 (UCS Diagnostic, Rome, Italy). Finally, samples were counteracted with 4′,6-diamidino-2-phenylindole (DAPI) counterstain (Kreatech Biotechnology B.V., Amsterdam, Netherlands).

A Nikon upright microscope ECLIPSE Ci–S (Nikon Corporation, Tokyo, Japan) connected to a Nikon digital camera was used to view the images, while the NIS-Elements software (5.30.01; Laboratory Imaging, Prague, Czech Republic) was used to capture them at 40 × magnification. The co-expression of NOX4 and SIRT1 in the different experimental conditions was measured by calculating the number of cells co-expressing NOX4 and SIRT1 relative to the total number of cells and expressed as a percentage.

### Alizarin red staining

Alizarin red staining was performed to detect the mineralization process. In accordance with previous studies [32], the cell samples were fixed with 4% paraformaldehyde for 15 min at the end of the experimental procedures. After washing with deionized H_2_O, the alizarin red solution (40 mm, pH 4.1) was added to each well. The plates were incubated at room temperature for 20 min with gentle agitation. Subsequently, excess dye was removed and four washes with abundant deionized H_2_O were performed. Images were acquired at 20 × magnification using NIS-Elements software (5.30.01; Laboratory Imaging, Prague, Czech Republic) and a Nikon ECLIPSE Ci–S upright microscope (Nikon Corporation, Tokyo, Japan) connected to a Nikon digital camera.

### Statistical analysis

All statistical analyses were conducted using GraphPad Prism 8 software (GraphPad Prism 8.0.1, La Jolla, CA, USA), expressing data as mean ± standard error. All data with a normal distribution were processed with Welch’s parametric test and were considered significantly different if *p* < 0.05.

## Results

### Clinical evaluation of the study population

The study population included a total of 20 male participants, divided into two groups based on clinical and instrumental evaluation: ten patients undergoing hip arthroplasty for osteoarthritis (OA) and ten patients undergoing hip arthroplasty for fragility fracture (OP).

Table 1 summarizes the different parameters analyzed for each patient, such as age (years), BMI (Kg/m^2^), *T*-score (L1–L4), *T*-score (femoral neck), and *T*-score (total femur). First, no discrepancies were found for age between OA (74.2 ± 1.2) and OP (76.1 ± 1.4) patients (*p* = 0.32), whereas a statistically significant difference was found for BMI values (OA: 25.5 ± 0.8 vs OP: 23.2 ± 0.5, *p* < 0.05). Interestingly, the assessment of BMD of the lumbar spine, femoral neck and total femur, expressed as *T*-score, showed statistically significant differences between the two experimental groups (*p* < 0.01). In fact, OA patients had *T*-score (L1–L4), *T*-score (femoral neck) and *T*-score (total femur) values of − 0.1 ± 0.5, − 1.0 ± 0.4 and − 1.5 ± 0.3, respectively. In contrast, values of − 1.8 ± 0.3, − 2.5 ± 0.2 and − 2.7 ± 0.1 were measured for *T*-score (L1–L4), *T*-score (femoral neck) and *T*-score (total femur), respectively, in the OP group.

**Table 1:** Clinical characteristics of OA and OP patients

Parameters	OA (*n* = 10)	OP (*n* = 10)	Significance
Age (years)	74.2 ± 1.2	76.1 ± 1.4	*p* = 0.32
BMI (Kg/m^2^)	25.5 ± 0.8	23.2 ± 0.5	*p* < 0.05
*T*-score (L1–L4)	− 0.1 ± 0.5	− 1.8 ± 0.3	*p* < 0.01
*T*-score (femoral neck)	− 1.0 ± 0.4	− 2.5 ± 0.2	*p* < 0.01
*T*-score (total femur)	− 1.5 ± 0.3	− 2.7 ± 0.1	*p* < 0.01

*OA* patients underwent hip arthroplasty for osteoarthritis, *OP* patients underwent hip arthroplasty for fragility fracture, *BMI* body mass index

### Effects of trolox, r-irisin and resveratrol cocktail treatment on osteoblast metabolism

Primary cultures of osteoblasts derived from OA and OP patients were treated with a cocktail of trolox, r-irisin and resveratrol for 6 days, evaluating their effects on cell viability, ROS levels and SA-β-gal activity.

**Fig. 1:**
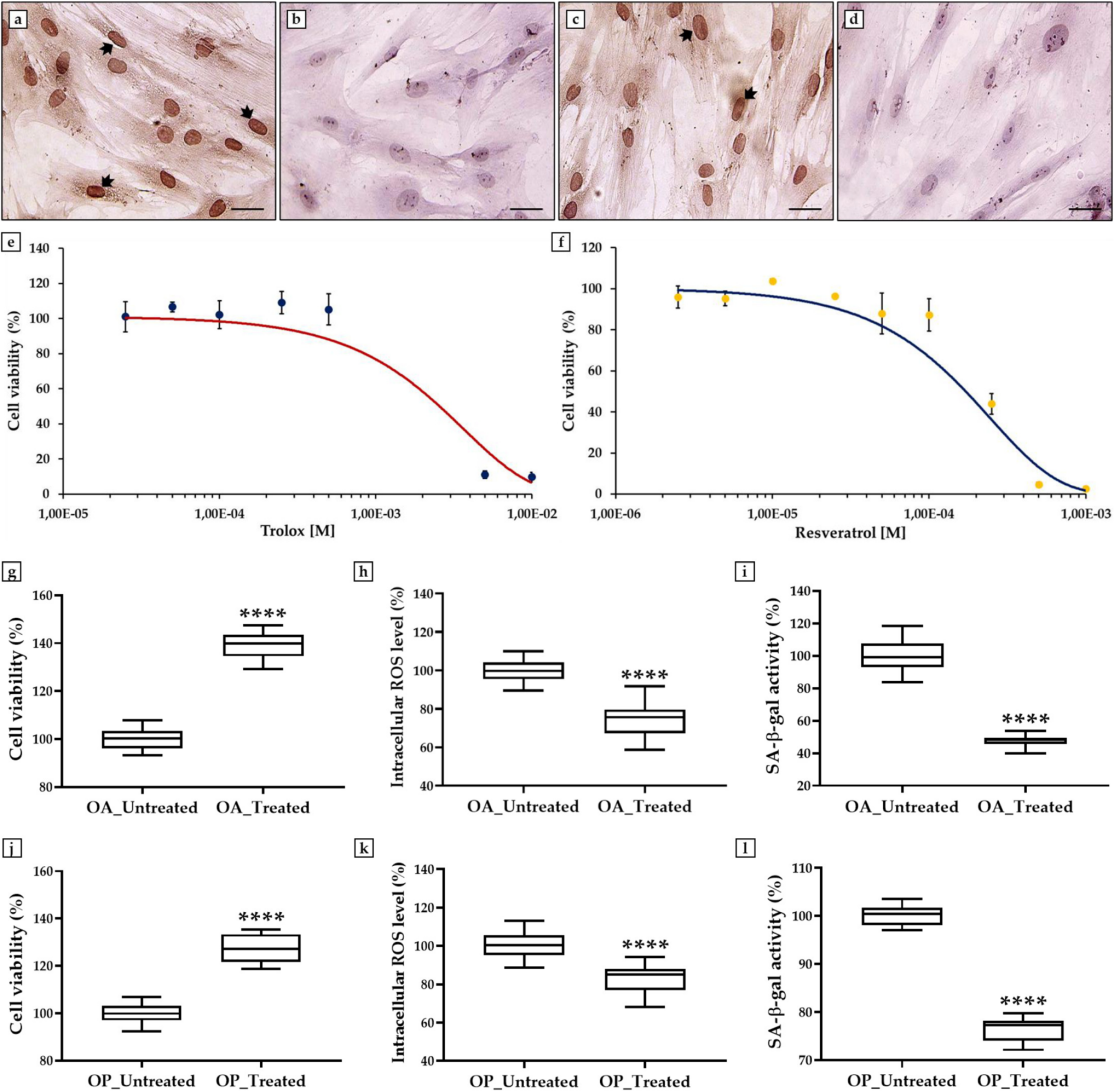
Effects of treatment with a cocktail of trolox, recombinant irisin (r-irisin), and resveratrol in osteoblasts from osteoarthritic (OA) and osteoporotic (OP) patients on cell viability, oxidative stress, and senescence-associated β-galactosidase (SA-β-gal) activity. **a**–**d** Immunocytochemistry for alkaline phosphatase (ALP): **a** OA osteoblasts incubated with anti-ALP antibody (arrows). **b** OA osteoblasts are used as a negative control. **c** OP osteoblasts incubated with anti-ALP antibody (arrows). **d** OP osteoblasts are used as a negative control. 40 × images, scale bar represents 100 μm. **e**, **f** Dose–response curves: the half inhibitory concentration (IC50) was obtained at a dosage between 2 × 10^–3^ M and 3 × 10^–3^ M for trolox and at a dosage about of 2 × 10^–4^ M for resveratrol. **g**, **j** MTS assay: a significant increase in cell viability was detected in treated cells of OA (*p* < 0.0001) and OP (*p* < 0.0001) patients compared with untreated cells (*n* = 25 from *N* = 5 experiments). **h**, **k** Intracellular reactive oxygen species (ROS) levels: a significant reduction in oxidative stress was detected in treated cells of OA and OP patients with respect to untreated cells (*p* < 0.0001) (*n* = 25 from *N* = 5 experiments). **i**, **l** SA-β-gal assay: a significant reduction in cell senescence was detected in treated cells of OA and OP patients with respect to untreated cells (*p* < 0.0001) (*n* = 15 from *N* = 5 experiments). Absorbance (**g**, **j**) and fluorescence (**h**, **k**, **i**, **l**) data were normalized relative to untreated cells, defined as 100%, for both OA and OP patients

First, an immunocytochemistry analysis was performed to characterize the cell cultures, assessing the expression of ALP, a specific marker associated with bone metabolism and osteoblastic differentiation [33]. Light microscopic analysis showed clear expression of ALP, confirming the osteoblastic phenotype and functional capabilities of these cells in both OA (Fig. 1a, b) and OP (Fig. 1c, d) patients.

Next, two dose–response curves were constructed to estimate the doses of trolox and resveratrol for which non-toxic effects are detected by treating cells with increasing concentrations of the two substances and then assessing cell viability by MTS assay. Regarding to r-irisin, the dosage of 10 ng/mL was used as recommended by the supplier and previously demonstrated [11, 30]. Figure 1e shows that treatment with trolox did not significantly affect cell viability up to a dosage of 2.5 × 10^–4^ M, whereas a progressive reduction was measured at higher concentrations of the substance. As half the inhibitory concentration (IC50) was obtained at a dosage between 2 × 10^–3^ M and 3 × 10^–3^ M, the cell cultures were treated with trolox at a concentration of 1 × 10^–4^ M. Relative to resveratrol, cell viability was not significantly affected by treatment up to a dosage of 5 × 10^–5^ M. In fact, Fig. 1f shows a progressive reduction in cell viability at higher concentrations of the substance, with an IC50 measured at a dose about of 2 × 10^–4^ M. Overlapping dose–response curves were obtained for OA and OP patients, so Fig. 1e, f shows the representative results obtained on primary osteoblast cultures from the OA group. Based on these results, osteoblastic cells were treated with resveratrol at a concentration of 2.5 × 10^–5^ M.

Interestingly, treatment of cells with the trolox, r-irisin and resveratrol cocktail promoted a significant increase in cell viability in both experimental conditions, with a greater increase in the OA group. In fact, the absorbance values measured by MTS assay in OA patients were 100.0 ± 0.8 in untreated cells and 138.9 ± 1.2 in treated cells (*p* < 0.0001) (Fig. 1g). Similarly, OP patients had absorbance values of 100.0 ± 0.8 in the absence of treatment and 127.1 ± 1.2 in the presence of treatment (*p* < 0.0001) (Fig. 1j).

In agreement, the measurement of intracellular ROS levels showed a significant reduction in oxidative stress after treatment with trolox, r-irisin and resveratrol cocktail compared to untreated cells. Specifically, intracellular ROS levels in the OA group were 100.0 ± 1.1 in untreated cells and 73.8 ± 1.7 in treated cells (*p* < 0.0001) (Fig. 1h), whereas intracellular ROS levels in the OP group were 100.0 ± 1.3 in untreated cells and 82.8 ± 1.5 in treated cells (*p* < 0.0001) (Fig. 1k).

Noteworthy, treatment with the trolox, r-irisin and resveratrol cocktail promoted a significant reduction in cell senescence especially in OA patients, where SA-β-gal activity levels were 100.0 ± 2.4 in untreated cells and 47.3 ± 1.0 in treated cells (*p* < 0.0001) (Fig. 1i). Similar results were also obtained in OP patients (*p* < 0.0001), as demonstrated by the significant reduction in SA-β-gal activity in treated cells (76.6 ± 0.6) compared to control cells (100.0 ± 0.5) (Fig. 1l).

### Effects of trolox, r-irisin and resveratrol cocktail treatment on NOX4 and SIRT1 expressions

Immunofluorescence and western blotting analyses were conducted to investigate any changes in the expression patterns of NOX4 and SIRT1 in primary cultures of osteoblasts treated with the trolox, r-irisin and resveratrol cocktail.

**Fig. 2:**
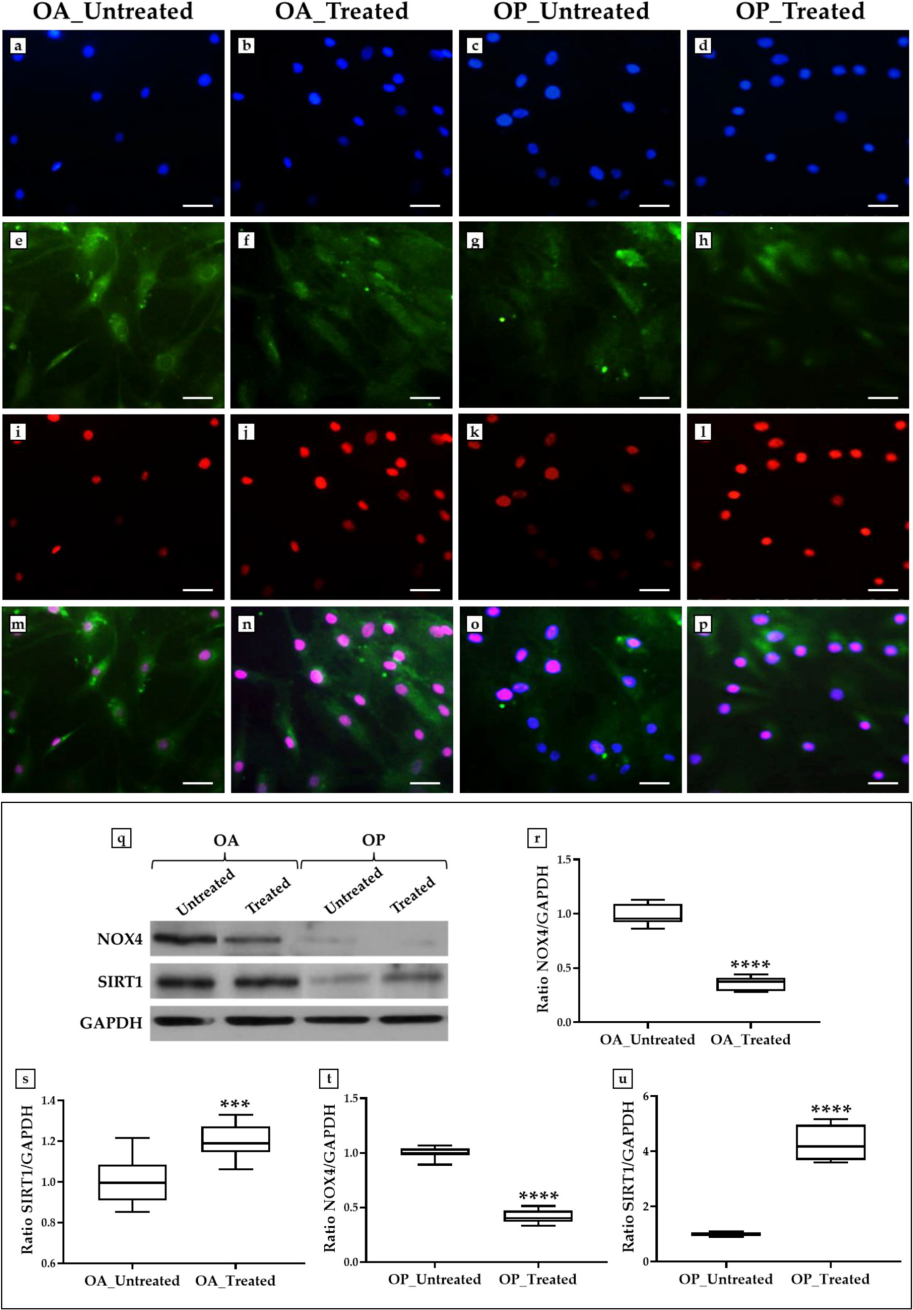
Analysis of NADPH Oxidase 4 (NOX4) and sirtuin 1 (SIRT1) expressions in primary cultures of osteoblasts from osteoarthritic (OA) and osteoporotic (OP) patients after treatment with a cocktail of trolox, recombinant irisin (r-irisin), and resveratrol. **a**–**p** Immunofluorescence: **a**–**d** nuclei are stained with DAPI (blue); **e**–**h** immunostaining for NOX4 (green); **i**–**l** immunostaining for SIRT1 (red); **m**–**p** merge for DAPI, NOX4 and SIRT1 signals. 40 × images, scale bar represents 100 μm. **q**–**u** Western blotting analysis: **r**, **t** highest expression of NOX4 was measured in the untreated cells of OA and OP patients with respect to treated cells (*p* < 0.0001). **s**, **u** Treatment with the cocktail of trolox, r-irisin and resveratrol promoted a significant increase in SIRT1 expression in both OA (*p* < 0.001) and OP (*p* < 0.0001) patients

Figure 2a–p highlights fluorescent signals for NOX4 and SIRT1 under all experimental conditions, although with marked differences between groups. Particularly, untreated cells from OA patients showed the highest co-expression of NOX4 and SIRT1 (Fig. 2m), with a percentage of cells co-expressing NOX4 and SIRT1 relative to the total number of cells of 87.6 ± 1.8. On the other hand, the percentage of cells co-expressing NOX4 and SIRT1 was 30.5 ± 2.9 in the OA_Treated group, 9.2 ± 1.6 in the OP_Untreated group, and 12.2 ± 2.3 in the OP_Treated group.

In agreement, western blotting analysis showed a positive band at about 67 kDa, corresponding to the molecular weight of NOX4, and a positive band at about 110 kDa, corresponding to the molecular weight of SIRT1, in the protein extracts of all cell samples, with higher amounts for cells derived from OA patients (Fig. 2q). Overall, the highest expression of NOX4 was measured in untreated cells from OA patients, while SIRT1 was more highly expressed in treated cells from the same group. In fact, the mean values of NOX4 expression obtained by densitometric analysis were 1.00 ± 0.03 in the OA_Untreated group and 0.35 ± 0.02 in the OA_Treated group (*p* < 0.0001) (Fig. 2r). On the other hand, the mean expression values of SIRT1 were 1.00 ± 0.04 in the OA_Untreated group and 1.20 ± 0.03 in the OA_Treated group (*p* < 0.001) (Fig. 2s).

Interestingly, treatment with the cocktail of trolox, r-irisin and resveratrol also affected the expression of NOX4 and SIRT1 in OP patients. In detail, the mean NOX4 expression values were 1.00 ± 0.02 in the OP_Untreated group and 0.41 ± 0.02 in the OP_Treated group (*p* < 0.0001) (Fig. 2t). In contrast, a significant increase in SIRT1 was observed after treatment, with mean expression values of 1.00 ± 0.02 in the OP_Untreated group and 4.34 ± 0.21 in the OP_Treated group (*p* < 0.0001) (Fig. 2u).

### Effects of trolox, r-irisin and resveratrol cocktail on mineralization process and PTX3 expression

The effects of treatment with the cocktail of trolox, r-irisin and resveratrol on the mineralization process were investigated by alizarin red staining. In addition, immunocytochemistry and western blotting analyses were performed to measure the expression of PTX3, a known regulator of bone metabolism [13].

**Fig. 3:**
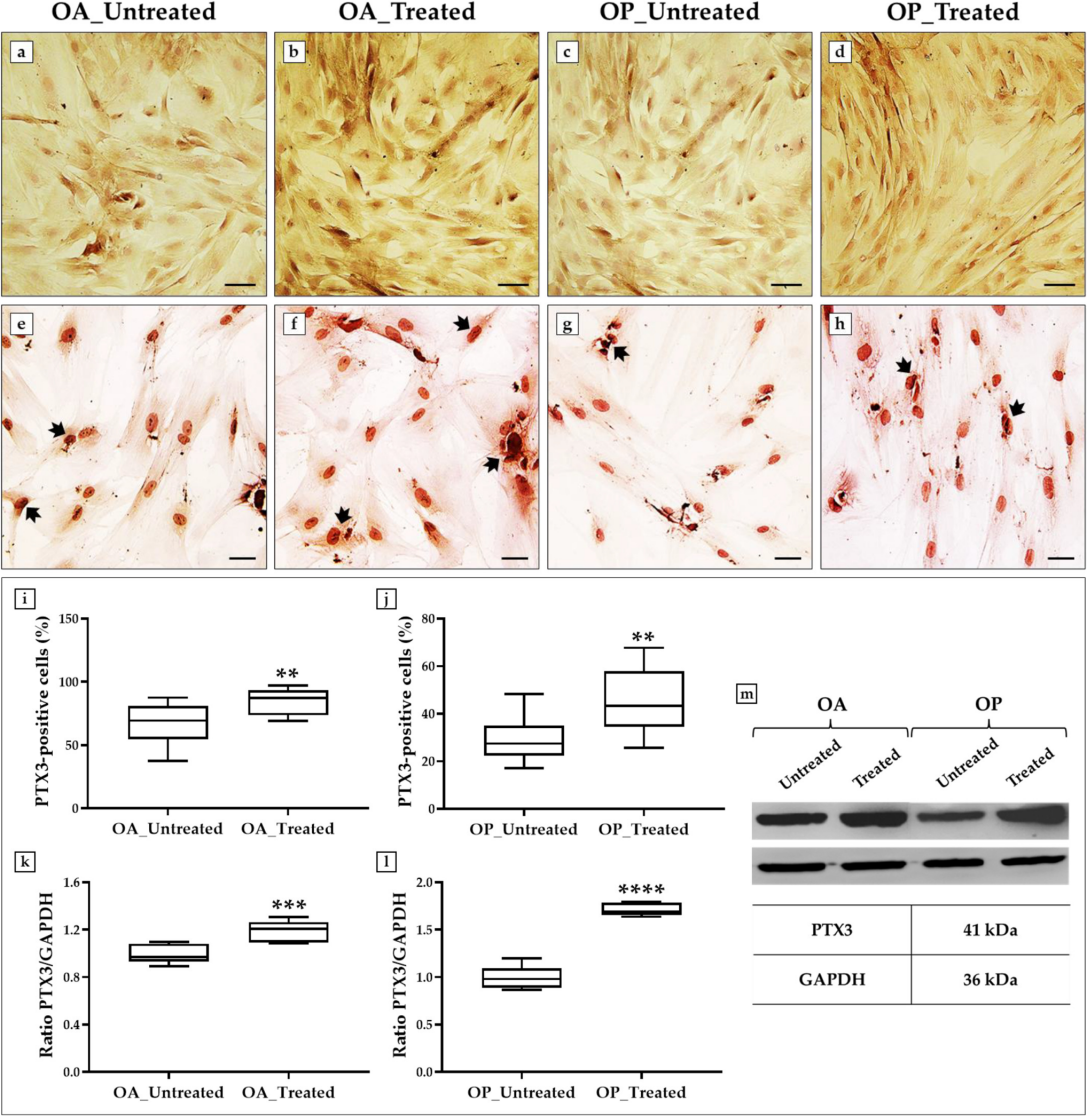
Alizarin red staining and analysis of pentraxin 3 (PTX3) expression in primary cultures of osteoblasts from osteoarthritic (OA) and osteoporotic (OP) patients after treatment with a cocktail of trolox, recombinant irisin (r-irisin), and resveratrol. **a**–**d** Alizarin red staining: the greatest mineral deposition was observed in the OA_Treated group, although the OP_Treated group also showed marked staining. 20 × images, scale bar represents 50 μm. **e**–**h** Immunocytochemistry: **e**, **f**, **i** highest expression of PTX3 (arrows) was observed in the OA-treated group compared with untreated cells (*p* < 0.01). **g**, **h**, **j** Treatment with trolox, r-irisin and resveratrol cocktail also promoted a significant increase in PTX3 expression (arrows) in the OP_Treated group with respect to untreated cells (*p* < 0.01) (*n* = 12 from *N* = 4 experiments). 40 × images, scale bar represents 100 μm. **k**–**m** Western blotting analysis: **k**, **m** higher expression of PTX3 (top line) was measured in treated cells of OA patients compared to untreated cells (*p* < 0.001). **l**, **m** Significant increase in PTX3 (top line) was detected in treated cells of OP patients compared with untreated cells (*p* < 0.0001) (*n* = 9 from *N* = 3 experiments)

Interestingly, greater mineral deposition was observed in the OA_Treated group, as evidenced by the more intense staining in Fig. 3b with respect to the untreated cells (Fig. 3a). The OP_Treated group also showed marked mineral deposition compared to untreated cells (Fig. 3c, d), although with a less pronounced effect than OA patients.

In agreement, immunocytochemical analysis showed the presence of PTX3 in all experimental conditions, revealing significant differences between groups. The results were expressed as the percentage of cells positive for PTX3 compared with the total analyzed. Notably, the highest levels of PTX3 were found in treated cells of OA patients, as the percentage of PTX3-positive cells was 66.3 ± 4.7 in the OA_Untreated group and 84.9 ± 2.9 in the OA_Treated group (*p* < 0.01) (Fig. 3e, f, i). Furthermore, treatment with the cocktail of trolox, r-irisin and resveratrol promoted a significant increase in protein expression even in OP patients. In fact, the percentage of PTX3-positive cells was 28.8 ± 2.5 in the OP_Untreated group and 45.3 ± 3.8 in the OP_Treated group (*p* < 0.01) (Fig. 3g, h, j).

These results were confirmed by western blotting analysis, which detected a positive band at about 41 kDa, corresponding to the molecular weight of PTX3, in all protein extracts analyzed. Again, the highest protein expression was observed in OA osteoblasts treated with the cocktail of trolox, r-irisin and resveratrol. In fact, densitometric analysis showed mean PTX3 expression values of 1.00 ± 0.03 in untreated cells and 1.19 ± 0.03 in treated cells (*p* < 0.001) (Fig. 3k, m). Similarly, a significant increase in PTX3 levels was detected in OP patients after treatment, with mean protein expression values of 1.00 ± 0.04 in untreated cells and 1.71 ± 0.02 in treated cells (*p* < 0.0001) (Fig. 3l, m).

## Discussion

Osteoarthritis and osteoporosis are age-related disease characterized by metabolic changes, such as increased oxidative stress and cellular senescence that affect the structure and function of bone tissue [34]. The therapeutic strategies available for the management of these disorders are mainly based on pharmacological treatment, as well as surgery, aimed at counteracting bone resorption, reducing musculoskeletal pain and improving mobility [35]. However, scientific research in this field is constantly evolving, exploring new molecular targets and drug combinations to counteract the development and progression of age-related diseases [36, 37]. In this context, some evidence has reported the effectiveness of trolox, r-irisin and resveratrol in attenuating alterations in osteoblast metabolism, although the effect of treatment with a cocktail of these substances has never been verified. Therefore, the aim of this study was to investigate the effects of combined treatment with trolox, r-irisin and resveratrol on the metabolism of osteoblasts isolated from patients undergoing hip arthroplasty for coxarthrosis or fragility fracture, assessing the potential modulation of key regulators of oxidative stress and mineralization, such as NOX4, SIRT1 and PTX3.

Quantitative investigations showed a significant increase in the viability of treated osteoblasts in both experimental groups, concomitant with a reduction in intracellular ROS and SA-β-Gal levels. Although no direct statistical comparison was made between OA and OP patients, the measured values indicate different responses. In fact, the increase in cell viability was more evident in the OA group, while lower baseline ROS levels were observed in the OP group in association with higher senescent activity. These data reflect the different pathophysiological characteristics of the two diseases, with osteoarthritis characterized by inflammatory processes that affect the bone microenvironment, while osteoporosis appears to present more advanced cellular senescence and reduced regenerative potential [38, 39]. Furthermore, western blotting analyses showed a down-regulation of NOX4, responsible for ROS production, as well as an up-regulation of SIRT1, a regulator of cell viability and senescence, and PTX3, known to be involved in osteogenesis and bone mineralization. However, although treatment with the cocktail positively influenced osteoblast metabolism, differences were found between groups, with effects being more pronounced in cells isolated from OA patients, suggesting a lower efficacy of the cocktail in the context of osteoporosis.

Our results agree with the observations of Chen et al., who found increased expression of osteogenic markers and improved differentiation in osteoblasts treated with r-irisin and exposed to simulated microgravity [40]. Similarly, Qiao and colleagues observed an up-regulation of runt-related transcription factor 2 (RUNX2) and osteocalcin in r-irisin-treated osteoblasts through activation of the P38/extracellular signal-regulated kinase (ERK) mitogen-activated protein kinase (MAPK) signaling pathway, confirming the ability of this molecule in promoting bone formation [41]. Noteworthy, the benefits of r-irisin on osteoblast metabolism could depend on an up-regulation of PTX3, as Cariati et al. demonstrated that r-irisin treatment preserves the expression of this marker in human osteoblasts exposed to simulated microgravity. In addition, an increase in cell viability and a reduction in oxidative stress were observed, suggesting a role for irisin as both osteogenic and anti-apoptotic [11]. In agreement, other authors have shown that increasing PTX3 expression in osteoblasts enhances hydroxyapatite crystal formation and stimulates the activity of key osteogenic markers, such as RUNX2 and ALP, confirming their involvement in bone formation and mineralization [42, 43]. Overall, r-irisin, through modulation of PTX3, could help improve osteoblastic function and bone matrix quality by promoting cell viability and reducing oxidative stress, suggesting its potential therapeutic role for the treatment of degenerative bone diseases.

Interestingly, an improvement in bone microarchitecture in mouse models subjected to vibratory training has recently been suggested in association with increased expression of fibronectin type III domain-containing protein 5 (FNDC5), the precursor of the hormone irisin, up-regulation of SIRT1 and down-regulation of NOX4, confirming the role of these markers in bone metabolism [44]. Indeed, SIRT1 is a key regulator of mitochondrial biogenesis, and its cytoplasmic depletion has been associated with the progression of osteoporosis and arthrosis in mouse models [45, 46]. On the other hand, NOX4 is known to promote osteoclastogenesis and bone loss, although some evidence points to its role in bone formation and osteoblastic differentiation, highlighting the need for further studies to clarify the function of this oxidase in bone pathophysiology [47, 48]. In fact, our results showed a different expression of NOX4 between the OA_Untreated and OP_Untreated groups, suggesting its involvement in alterations in osteoblastic metabolism. Although treatment reduced NOX4 expression in both experimental groups, further investigation is needed to assess the influence of the cocktail on protein synthesis and degradation processes. Nevertheless, the development of strategies with antioxidant action capable of counteracting ROS over-production and promoting SIRT1 activation could attenuate the alterations in osteoblastic metabolism that occur with aging. In this regard, the potent antioxidant action of trolox, in association with the presence of resveratrol, could lead to a modulation of the NOX4–SIRT1 axis, with effects on cell viability, oxidative stress and cellular senescence. Not surprisingly, Mody et al. demonstrated that trolox effectively counteracts H_2_O_2_-induced inhibition of osteogenic markers in osteoblastic MC3T3-E1 cells, suggesting a role for antioxidants in enhancing bone cell differentiation by reducing basal levels of ROS [49]. In addition, several evidences support the effectiveness of resveratrol in improving bone homeostasis as, due to its multiple actions on both osteoblasts and osteoclasts, it is known to improve trabecular microarchitecture and mitigate the loss of bone mass associated with aging [50–52].

Overall, age-related musculoskeletal diseases, such as osteoarthritis and osteoporosis, are characterized by a complex pathophysiology and the involvement of different pathways and molecular targets that differentially participate in alterations of osteoblastic metabolism. The administration of a cocktail consisting of trolox, r-irisin and resveratrol could represent an innovative strategy to counteract these alterations, improving cell viability and osteogenic potential and reducing the levels of intracellular ROS and SA-β-Gal. However, further studies are needed to investigate the efficacy of this cocktail, as well as to evaluate the use of therapies based on the combination of several compounds capable of influencing osteoblast metabolism during aging.

### Limits of study

This pilot study aimed to investigate the effects of a cocktail with trolox, r-irisin and resveratrol on the metabolism of osteoblasts isolated from patients with coxarthrosis or fragility fracture by assessing the modulation of NOX4, SIRT1 and PTX3. Although our results may represent a starting point for the development of innovative therapies to counteract the metabolic alterations underlying osteoarthritis and osteoporosis, some limitations need to be discussed. First, this study represents a preliminary evaluation based on the enrolment of 20 patients divided into two groups. Despite the interesting results on the effects of trolox, r-irisin and resveratrol, our results will have to be confirmed by further studies with larger patient cohorts to verify the involvement of additional pathways and molecular targets potentially modulated by the cocktail used. Furthermore, although primary cultures of osteoblasts were used to study treatment responses, investigations in animal models will be necessary to identify optimal dosages and verify the effect in vivo.

### Conclusions

Osteoarthritis and osteoporosis are diseases characterized by deep alterations in osteoblast metabolism, such as reduced cell viability, as well as increased oxidative stress and cell senescence. Importantly, NOX4, SIRT1 and PTX3 represent important molecular players involved in these processes, significantly affecting bone structural integrity and mineralization. Pharmacological modulation of these factors, through the administration of a cocktail of trolox, r-irisin and resveratrol, could lay the foundations for new forms of management of bone diseases characterized by altered osteoblast metabolism. Undoubtedly, the development of pharmacological combinations capable of modulating the expression of multiple molecular targets and influencing different cellular processes represents an important frontier in regenerative medicine and offers the opportunity to tailor treatment, in terms of composition and dosage, to the patient’s individual needs.

## Supplementary Information

Below is the link to the electronic supplementary material.

Supplementary file1 (DOCX 508 KB)

## Data Availability

The original contributions presented in the study are included in the article/Supplementary Material, further inquiries can be directed to the corresponding author.

## References

[1] Falvino A, Bonanni R, Tarantino U et al (2025) Which approach to choose to counteract musculoskeletal aging? A comprehensive review on the multiple effects of exercise. Int J Mol Sci. 10.3390/ijms26157573 40806700 10.3390/ijms26157573 PMC12347637

[2] López-Otín C, Blasco MA, Partridge L et al (2023) Hallmarks of aging: an expanding universe. Cell 186:243–278. 10.1016/j.cell.2022.11.001 36599349 10.1016/j.cell.2022.11.001

[3] Tarantino U, Visconti VV, Bonanni R et al (2022) Osteosarcopenia and long-COVID: a dangerous combination. Ther Adv Musculoskelet Dis 14:1759720X221130485. 10.1177/1759720X221130485 36317068 10.1177/1759720X221130485 PMC9614591

[4] Bultink IEM, Lems WF (2013) Osteoarthritis and osteoporosis: what is the overlap? Curr Rheumatol Rep 15:328. 10.1007/s11926-013-0328-0 23508809 10.1007/s11926-013-0328-0

[5] Geusens PP, van den Bergh JP (2016) Osteoporosis and osteoarthritis: shared mechanisms and epidemiology. Curr Opin Rheumatol 28:97–103. 10.1097/BOR.0000000000000256 26780427 10.1097/BOR.0000000000000256

[6] Zhao R, Chen Y, Wang D et al (2023) Role of irisin in bone diseases. Front Endocrinol (Lausanne) 14:1212892. 10.3389/fendo.2023.1212892 37600697 10.3389/fendo.2023.1212892 PMC10436578

[7] Hu X, Wang Z, Wang W et al (2024) Irisin as an agent for protecting against osteoporosis: a review of the current mechanisms and pathways. J Adv Res 62:175–186. 10.1016/j.jare.2023.09.001 37669714 10.1016/j.jare.2023.09.001 PMC11331170

[8] Ostojic M, Zevrnja A, Vukojevic K, Soljic V (2021) Immunofluorescence analysis of NF-kB and iNOS expression in different cell populations during early and advanced knee osteoarthritis. Int J Mol Sci. 10.3390/ijms22126461 34208719 10.3390/ijms22126461 PMC8233870

[9] Zhang J, Valverde P, Zhu X et al (2017) Exercise-induced irisin in bone and systemic irisin administration reveal new regulatory mechanisms of bone metabolism. Bone Res 5:16056. 10.1038/boneres.2016.56 28944087 10.1038/boneres.2016.56 PMC5605767

[10] Storlino G, Colaianni G, Sanesi L et al (2020) Irisin prevents disuse-induced osteocyte apoptosis. J Bone Miner Res 35:766–775. 10.1002/jbmr.3944 31826311 10.1002/jbmr.3944

[11] Cariati I, Bonanni R, Rinaldi AM et al (2023) Recombinant irisin prevents cell death and mineralization defects induced by random positioning machine exposure in primary cultures of human osteoblasts: a promising strategy for the osteoporosis treatment. Front Physiol. 10.3389/fphys.2023.1107933 37008023 10.3389/fphys.2023.1107933 PMC10052411

[12] Parente R, Sobacchi C, Bottazzi B et al (2019) The long pentraxin PTX3 in bone homeostasis and pathology. Front Immunol 10:2628. 10.3389/fimmu.2019.02628 31787987 10.3389/fimmu.2019.02628 PMC6856142

[13] Cariati I, Bonanni R, Scimeca M et al (2022) Exposure to random positioning machine alters the mineralization process and PTX3 expression in the SAOS-2 cell line. Life. 10.3390/life12050610 35629278 10.3390/life12050610 PMC9143356

[14] Atiq A, Lee HJ, Khan A et al (2023) Vitamin E analog trolox attenuates MPTP-induced Parkinson’s disease in mice, mitigating oxidative stress, neuroinflammation, and motor impairment. Int J Mol Sci. 10.3390/ijms24129942 37373089 10.3390/ijms24129942 PMC10298414

[15] Morabito C, Guarnieri S, Cucina A et al (2020) Antioxidant strategy to prevent simulated microgravity-induced effects on bone osteoblasts. Int J Mol Sci. 10.3390/ijms21103638 32455731 10.3390/ijms21103638 PMC7279347

[16] Lee J-H, Kim H-N, Yang D et al (2009) Trolox prevents osteoclastogenesis by suppressing RANKL expression and signaling. J Biol Chem 284:13725–13734. 10.1074/jbc.M806941200 19299513 10.1074/jbc.M806941200 PMC2679474

[17] Deng J, Ouyang P, Li W et al (2021) Curcumin alleviates the senescence of canine bone marrow mesenchymal stem cells during in vitro expansion by activating the autophagy pathway. Int J Mol Sci. 10.3390/ijms222111356 34768788 10.3390/ijms222111356 PMC8583405

[18] Geng Q, Gao H, Yang R et al (2019) Pyrroloquinoline quinone prevents estrogen deficiency-induced osteoporosis by inhibiting oxidative stress and osteocyte senescence. Int J Biol Sci 15:58–68. 10.7150/ijbs.25783 30662347 10.7150/ijbs.25783 PMC6329928

[19] Cao Z, Liu G, Zhang H et al (2022) Nox4 promotes osteoblast differentiation through TGF-beta signal pathway. Free Radic Biol Med 193:595–609. 10.1016/j.freeradbiomed.2022.11.016 36372285 10.1016/j.freeradbiomed.2022.11.016

[20] Zhang H, Wang A, Li G et al (2023) Osteoporotic bone loss from excess iron accumulation is driven by NOX4-triggered ferroptosis in osteoblasts. Free Radic Biol Med 198:123–136. 10.1016/j.freeradbiomed.2023.01.026 36738798 10.1016/j.freeradbiomed.2023.01.026

[21] Wegner AM, Haudenschild DR (2020) NADPH oxidases in bone and cartilage homeostasis and disease: a promising therapeutic target. J Orthop Res Off Publ Orthop Res Soc 38:2104–2112. 10.1002/jor.24693 10.1002/jor.24693 32285964

[22] Chen Y, Zhou F, Liu H et al (2021) SIRT1, a promising regulator of bone homeostasis. Life Sci 269:119041. 10.1016/j.lfs.2021.119041 33453243 10.1016/j.lfs.2021.119041

[23] Zhou L, Il WS, Moon YJ et al (2017) Overexpression of SIRT1 prevents hypoxia-induced apoptosis in osteoblast cells. Mol Med Rep 16:2969–2975. 10.3892/mmr.2017.6917 28677728 10.3892/mmr.2017.6917

[24] Chen Y, Xiao H, Liu Z et al (2024) Sirt1: an increasingly interesting molecule with a potential role in bone metabolism and osteoporosis. Biomolecules. 10.3390/biom14080970 39199358 10.3390/biom14080970 PMC11352324

[25] Ciccone L, Piragine E, Brogi S et al (2022) Resveratrol-like compounds as SIRT1 activators. Int J Mol Sci. 10.3390/ijms232315105 36499460 10.3390/ijms232315105 PMC9738298

[26] Yang X, Jiang T, Wang Y, Guo L (2019) The role and mechanism of SIRT1 in Resveratrol-regulated osteoblast autophagy in osteoporosis rats. Sci Rep 9:18424. 10.1038/s41598-019-44766-3 31804494 10.1038/s41598-019-44766-3 PMC6895060

[27] Wong RH, Thaung Zaw JJ, Xian CJ, Howe PR (2020) Regular supplementation with resveratrol improves bone mineral density in postmenopausal women: a randomized, placebo-controlled trial. J Bone Miner Res 35:2121–2131. 10.1002/jbmr.4115 32564438 10.1002/jbmr.4115 PMC7689937

[28] Kellgren JH, Lawrence JS (1957) Radiological assessment of osteo-arthrosis. Ann Rheum Dis 16:494–502. 10.1136/ard.16.4.494 13498604 10.1136/ard.16.4.494 PMC1006995

[29] Belfiore M, Cariati I, Matteucci A et al (2019) Calcitonin native prefibrillar oligomers but not monomers induce membrane damage that triggers NMDA-mediated Ca(2+)-influx, LTP impairment and neurotoxicity. Sci Rep 9:5144. 10.1038/s41598-019-41462-0 30914688 10.1038/s41598-019-41462-0 PMC6435710

[30] Bonanni R, Cariati I, Rinaldi AM et al (2024) Trolox and recombinant Irisin as a potential strategy to prevent neuronal damage induced by random positioning machine exposure in differentiated HT22 cells. PLoS ONE 19:e0300888. 10.1371/journal.pone.0300888 38512830 10.1371/journal.pone.0300888 PMC10956770

[31] Bonanni R, Falvino A, Matticari A et al (2025) Targeting ERRs to counteract age-related muscle atrophy associated with physical inactivity: a pilot study. Front Physiol 16:1616693. 10.3389/fphys.2025.1616693 40692696 10.3389/fphys.2025.1616693 PMC12277287

[32] Gregory CA, Gunn WG, Peister A, Prockop DJ (2004) An alizarin red-based assay of mineralization by adherent cells in culture: comparison with cetylpyridinium chloride extraction. Anal Biochem 329:77–84. 10.1016/j.ab.2004.02.002 15136169 10.1016/j.ab.2004.02.002

[33] Fan S, Zhang C, Sun X et al (2024) Metformin enhances osteogenic differentiation of BMSC by modulating macrophage M2 polarization. Sci Rep 14:20267. 10.1038/s41598-024-71318-1 39217251 10.1038/s41598-024-71318-1 PMC11365931

[34] Im G-I, Kim M-K (2014) The relationship between osteoarthritis and osteoporosis. J Bone Miner Metab 32:101–109. 10.1007/s00774-013-0531-0 24196872 10.1007/s00774-013-0531-0

[35] Gezer HH, Ostor A (2023) What is new in pharmacological treatment for osteoarthritis? Best Pract Res Clin Rheumatol 37:101841. 10.1016/j.berh.2023.101841 37302928 10.1016/j.berh.2023.101841

[36] Song S, Guo Y, Yang Y, Fu D (2022) Advances in pathogenesis and therapeutic strategies for osteoporosis. Pharmacol Ther 237:108168. 10.1016/j.pharmthera.2022.108168 35283172 10.1016/j.pharmthera.2022.108168

[37] Zhang Y, Ma J, Zhang W (2021) Berberine for bone regeneration: therapeutic potential and molecular mechanisms. J Ethnopharmacol 277:114249. 10.1016/j.jep.2021.114249 34058315 10.1016/j.jep.2021.114249

[38] Bai R-J, Li Y-S, Zhang F-J (2022) Osteopontin, a bridge links osteoarthritis and osteoporosis. Front Endocrinol (Lausanne) 13:1012508. 10.3389/fendo.2022.1012508 36387862 10.3389/fendo.2022.1012508 PMC9649917

[39] He X, Hu W, Zhang Y et al (2023) Cellular senescence in skeletal disease: mechanisms and treatment. Cell Mol Biol Lett 28:88. 10.1186/s11658-023-00501-5 37891477 10.1186/s11658-023-00501-5 PMC10612178

[40] Chen Z, Zhang Y, Zhao F et al (2020) Recombinant irisin prevents the reduction of osteoblast differentiation induced by stimulated microgravity through increasing β-catenin expression. Int J Mol Sci. 10.3390/ijms21041259 32070052 10.3390/ijms21041259 PMC7072919

[41] Qiao X, Nie Y, Ma Y et al (2016) Irisin promotes osteoblast proliferation and differentiation via activating the MAP kinase signaling pathways. Sci Rep 6:18732. 10.1038/srep18732 26738434 10.1038/srep18732 PMC4704023

[42] Grčević D, Sironi M, Valentino S et al (2018) The long pentraxin 3 plays a role in bone turnover and repair. Front Immunol 9:417. 10.3389/fimmu.2018.00417 29556234 10.3389/fimmu.2018.00417 PMC5845433

[43] Liu Y, Wang H, Zhou X-Z et al (2020) Pentraxin 3 promotes the osteoblastic differentiation of MC3T3-E1 cells through the PI3K/Akt signaling pathway. Biosci Rep. 10.1042/BSR20201165 10.1042/BSR20201165 PMC7284320 32436939

[44] Cariati I, Bonanni R, Romagnoli C et al (2025) Bone adaptations to a whole body vibration protocol in murine models of different ages: a preliminary study on structural changes and biomarker evaluation. J Funct Morphol Kinesiol. 10.3390/jfmk10010026 39846667 10.3390/jfmk10010026 PMC11755639

[45] Zainabadi K, Liu CJ, Caldwell ALM, Guarente L (2017) SIRT1 is a positive regulator of in vivo bone mass and a therapeutic target for osteoporosis. PLoS ONE 12:e0185236. 10.1371/journal.pone.0185236 28937996 10.1371/journal.pone.0185236 PMC5609767

[46] Matsuzaki T, Matsushita T, Takayama K et al (2014) Disruption of Sirt1 in chondrocytes causes accelerated progression of osteoarthritis under mechanical stress and during ageing in mice. Ann Rheum Dis 73:1397–1404. 10.1136/annrheumdis-2012-202620 23723318 10.1136/annrheumdis-2012-202620

[47] Goettsch C, Babelova A, Trummer O et al (2013) NADPH oxidase 4 limits bone mass by promoting osteoclastogenesis. J Clin Invest 123:4731–4738. 10.1172/JCI67603 24216508 10.1172/JCI67603 PMC3809780

[48] Chen J-R, Lazarenko OP, Blackburn ML et al (2022) Nox4 expression in osteo-progenitors controls bone development in mice during early life. Commun Biol 5:583. 10.1038/s42003-022-03544-0 35701603 10.1038/s42003-022-03544-0 PMC9198054

[49] Mody N, Parhami F, Sarafian TA, Demer LL (2001) Oxidative stress modulates osteoblastic differentiation of vascular and bone cells. Free Radic Biol Med 31:509–519. 10.1016/s0891-5849(01)00610-4 11498284 10.1016/s0891-5849(01)00610-4

[50] Tou JC (2015) Resveratrol supplementation affects bone acquisition and osteoporosis: pre-clinical evidence toward translational diet therapy. Biochim Biophys Acta 1852:1186–1194. 10.1016/j.bbadis.2014.10.003 25315301 10.1016/j.bbadis.2014.10.003

[51] Corbi G, Nobile V, Conti V et al (2023) Equol and resveratrol improve bone turnover biomarkers in postmenopausal women: a clinical trial. Int J Mol Sci 24:12063. 10.3390/ijms241512063 37569440 10.3390/ijms241512063 PMC10419295

[52] Wang X, Lu C, Chen Y et al (2023) Resveratrol promotes bone mass in ovariectomized rats and the SIRT1 rs7896005 SNP is associated with bone mass in women during perimenopause and early postmenopause. Climacteric 26:25–33. 10.1080/13697137.2022.2073809 35674253 10.1080/13697137.2022.2073809

