# Supplementary material for: Trolox, r-irisin and resveratrol cocktail to counteract osteoblast metabolism alterations in osteoarthritis and osteoporosis
Source: J Bone Miner Metab. 2025 Sep 16;43(6):672–84. doi: 10.1007/s00774-025-01642-7 (PMC12701872; doi:10.1007/s00774-025-01642-7)
Supplement: Supplementary file 1 — Supplementary file1 (DOCX 508 KB) [file 774_2025_1642_MOESM1_ESM.docx]

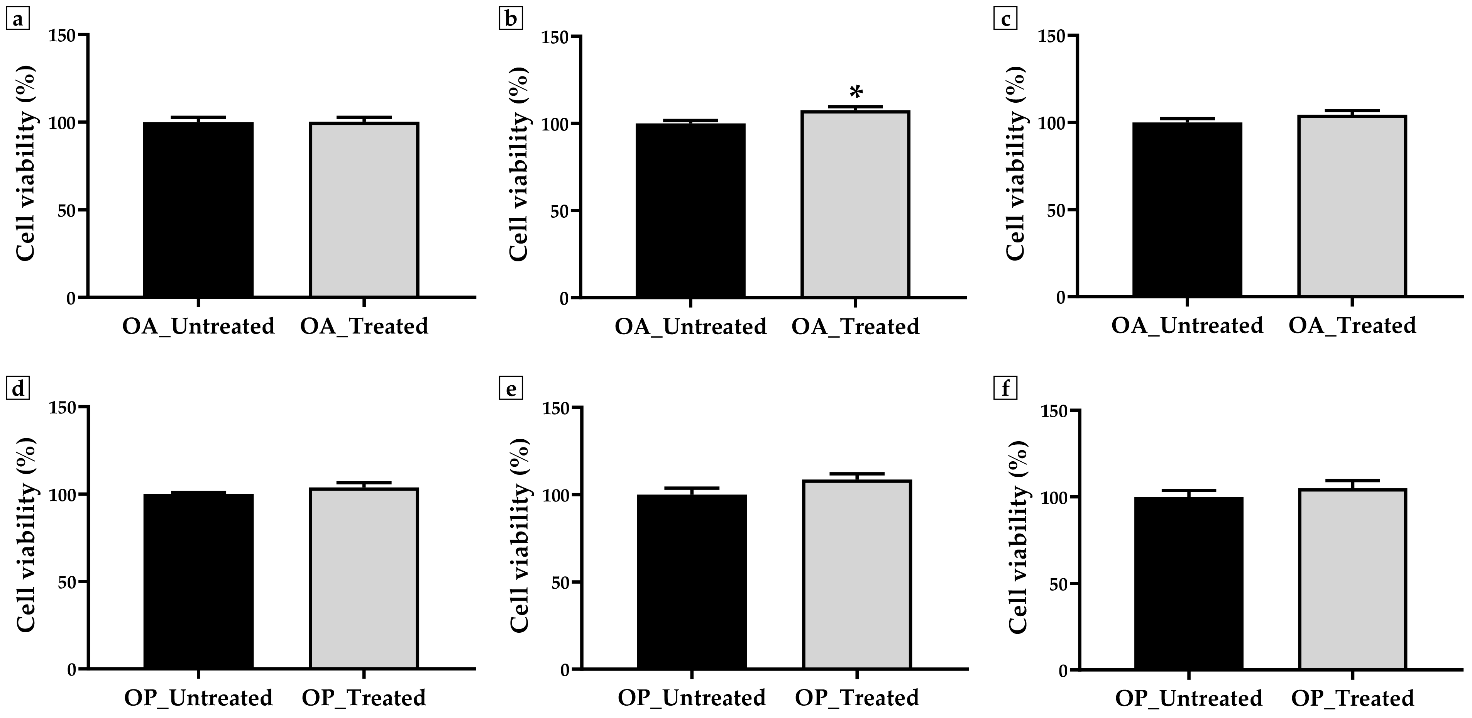


**Figure S1. Effects of treatment with a trolox, recombinant irisin (r-irisin), or resveratrol in osteoblasts from osteoarthritic (OA) and osteoporotic (OP) patients on cell viability.** (a) The absorbance values measured by MTS assay after trolox treatment were 100.0 ± 2.7 in the OA_Untreated group and 100.2 ± 2.5 in the OA_Treated group (p=0.9530). (b) The absorbance values measured by MTS assay after r-irisin treatment were 100.0 ± 1.7 in the OA_Untreated group and 107.6 ± 1.9 in the OA_Treated group (*p<0.01). (c) The absorbance values measured by MTS assay after resveratrol treatment were 100.0 ± 2.2 in the OA_Untreated group and 104.4 ± 2.4 in the OA_Treated group (p=0.2026). (d) The absorbance values measured by MTS assay after trolox treatment were 100.0 ± 0.9 in the OP_Untreated group and 103.8 ± 2.7 in the OP_Treated group (p=0.2261). (e) The absorbance values measured by MTS assay after r-irisin treatment were 100.0 ± 3.7 in the OP_Untreated group and 108.7 ± 3.3 in the OP_Treated group (p=0.1095). (f) The absorbance values measured by MTS assay after resveratrol treatment were 100.0 ± 3.7 in the OP_Untreated group and 104.9 ± 4.4 in the OP_Treated group (p=0.4149). Absorbance data were normalized relative to untreated cells, defined as 100%, for both OA and OP patients.


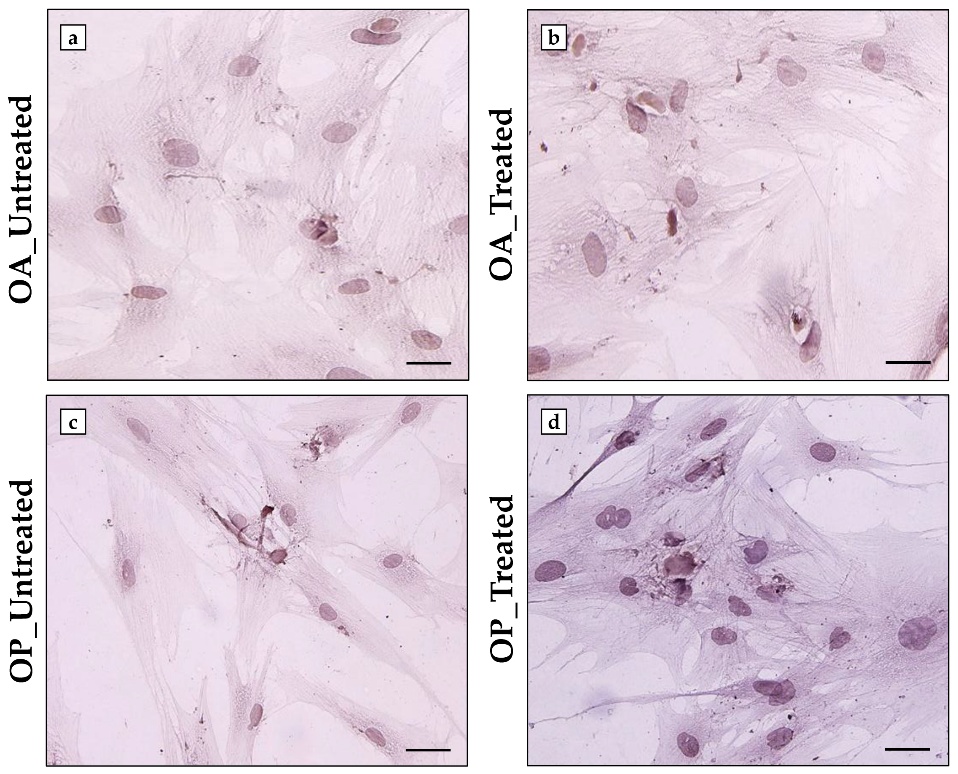


**Figure S2. Immunohistochemistry analysis for pentraxin 3 (PTX3) expression in primary cultures of osteoblasts from osteoarthritic (OA) and osteoporotic (OP) patients.** (a) PTX3-negative control in untreated osteoblasts from OA patients. (b) PTX3-negative control in treated osteoblasts from OA patients. (c) PTX3-negative control in untreated osteoblasts from OP patients. (d) PTX3-negative control in treated osteoblasts from OP patients. For 40× images, scale bar represents 50 μm.

**
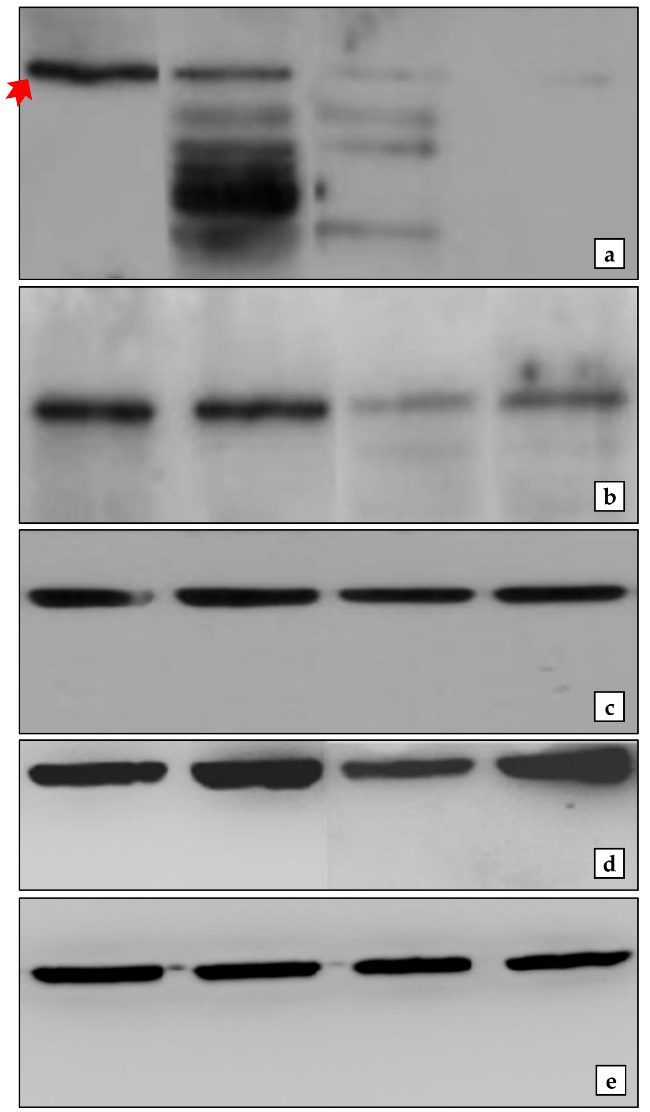
**

**Figure S3. Original western blotting images.** (a) NADPH Oxidase 4 (NOX4) expression (arrow, molecular weight 67 kDa) in osteoblasts from osteoarthritic (OA) and osteoporotic (OP) patients. (b) Sirtuin 1 (SIRT1) expression (molecular weight 110 kDa) in osteoblasts from OA and OP patients. (c) GAPDH expression (molecular weight 36 kDa) in osteoblasts from OA and OP patients used as housekeeping for NOX4 and SIRT1. (d) Pentraxin 3 (PTX3) expression (molecular weight 41 kDa) in osteoblasts from OA and OP patients. (e) GAPDH expression (molecular weight 36 kDa) in osteoblasts from OA and OP patients used as housekeeping for PTX3.
